# Nystagmus in adult patients with acute otitis media or otitis media with effusion without dizziness

**DOI:** 10.1371/journal.pone.0250357

**Published:** 2021-05-13

**Authors:** Chang-Hee Kim, Jiyeon Lee, BoYoon Choi, Jung Eun Shin

**Affiliations:** Department of Otorhinolaryngology-Head and Neck Surgery, Konkuk University Medical Center, Research Institute of Medical Science, Konkuk University School of Medicine, Seoul, Republic of Korea; University of Colorado Denver - Anschutz Medical Campus, UNITED STATES

## Abstract

The present study aimed to investigate the incidence and patterns of nystagmus in adult patients with acute otitis media (AOM) or otitis media with effusion (OME) without dizziness or vertigo, and discuss possible mechanisms. From February 2018 to November 2018, 34 consecutive patients with AOM or OME without dizziness were included. Nystagmus was examined with video Frenzel glasses. Of 34 adult AOM or OME patients without dizziness, nystagmus was observed in 28 patients (82%). In unilateral AOM or OME (*n* = 30), the most commonly observed nystagmus pattern was irritative-type direction-fixed nystagmus (*n* = 13), followed by paretic-type direction-fixed nystagmus (*n* = 8), and direction-changing positional nystagmus (*n* = 4). In bilateral AOM or OME (*n* = 4), direction-fixed nystagmus and direction-changing positional nystagmus were observed in two and one patients, respectively. Nystagmus was observed in as many as 82% of adult AOM or OME patients even though they did not complain of dizziness, and the pattern of nystagmus was either direction-fixed or direction-changing. Direct effect of inflammatory mediators penetrated from the middle ear and biochemical alteration in the inner ear fluids due to blood-perilymph barrier dysfunction may result in the presence of nystagmus in AOM or OME patients without dizziness.

## Introduction

Otitis media with effusion (OME) is defined as the presence of fluid in the middle ear cavity without signs or symptoms of acute ear infection, and, in contrast, acute otitis media (AOM) is defined as the rapid onset of signs and symptoms of inflammation in the middle ear, most often with ear pain and a bulging eardrum [1]. Inflammation in the middle ear may spread into the inner ear by penetrating the natural defensive barriers such as the oval window and round window membrane [2, 3], which may result in symptoms of inner ear dysfunction such as sensorineural hearing loss and dizziness [4, 5].

While it has been reported that OME and AOM can cause balance disorders and have been considered the most common cause of vestibular disturbances and vertigo in pediatric patients [6–21], the effect of OME or AOM on vestibular function has not been thoroughly investigated in adults patients with same disease category. Findings of vestibular dysfunction including bithermal caloric results, spontaneous and positional nystagmus have been reported in limited number of adult AOM patients with vertigo [3, 22], and the presence of dizziness or vertigo is considered indicative of one of the intratemporal complications of AOM. However, because dizziness is a subjective symptom defined as “a sensation of unsteadiness accompanied by a feeling of movement within the head” [23], the symptom threshold of dizziness may depend on individual’s sensitivity. Thus, patients with OME or AOM complicated by mild vestibular disturbance may not complain of dizziness symptom.

This study aimed to identify the presence of spontaneous or positional nystagmus in adult OME or AOM patients without dizziness.

## Methods

### Subjects

We evaluated 34 consecutive adult OME (*n* = 21) or AOM (*n* = 13) patients without dizziness (ten men and twenty-four women; mean age = 54 years; age range = 25–78 years) who visited Konkuk University Medical center, which is a tertiary referral center, from February 2018 to November 2018. Two AOM patients complaining of dizziness during the same period were excluded from the study. Male to female ratio was 7: 14 and 4: 9 in OME and AOM, respectively. OME was diagnosed when the patients had effusion in the middle ear without signs or symptoms of acute ear infection, and AOM was diagnosed when the patients showed middle ear effusion with acute onset and rapid progression of signs and symptoms of middle ear inflammation such as otalgia, and bulging or hyperemic tympanic membrane [1]. Because dizziness may mean different sensation to different people, the absence of dizziness symptom was confirmed by asking the patients if they feel lightheaded, have a swimming sensation in the head, have a sense of disorientation, imbalance or unsteadiness, and have an illusory sensation of motion of either the self or the surroundings [24]. The patients reported that they did not take medicine, which may elicit nystagmus, and they did not have a history of congenital nystagmus. Because the main purpose of the present study was to investigate the pattern of nystagmus in patients with OME or AOM without dizziness and the comparison of treatment outcome was out of this study’s scope, the present study was initiated without calculating the sample size.

For the comparison, thirty-four age- and sex-matched controls, who had no symptom of dizziness, or history of neurologic or otologic disorders, were recruited with written informed consent. The control subjects were selected from the patients, who visited our outpatient clinic with symptoms including parotid mass, thyroid mass or sore throat, and spontaneous and positional nystagmus were examined.

### Examination of nystagmus and laboratory tests

Spontaneous and positional nystagmus were examined at various head positions and recorded using goggles installed with an infrared camera. Spontaneous nystagmus was examined in a sitting position. A head-roll test was performed by turning the patient’s head to the right or to the left ~90° in a supine position. The patients also underwent a bow and lean test in a seated position by bending their heads forward to 90° and subsequently tilting their heads backward to 60°. Nystagmus finding was classified into two patterns; (1) Direction-fixed nystagmus when the direction of spontaneous nystagmus is not changed by positioning maneuvers, and (2) Direction-changing positional nystagmus when the direction of nystagmus was changed from that of spontaneous nystagmus by positioning maneuver. A bithermal caloric test was performed in two patients using an infrared video-based system (CHARTR VNG, ICS Medical). By irrigating the external ear canal with warm (44°C) and cool (30°C) for 30 s, the maximal slow-phase velocity of nystagmus was measured to calculate a canal paresis. The caloric test was conducted after the middle ear effusion was cleared with treatment, and a canal paresis of 25% or greater was considered abnormal. The hearing loss was evaluated with an average pure tone threshold at 5 frequencies (250, 500, 1000, 2000, and 4000 Hz) at the day of initial visit. The pattern of audiogram was categorized into conductive hearing loss and mixed hearing loss according to air- and bone-conduction threshold in pure tone audiometry. Determination of hearing level was made with respect to that of the opposite ear in unilateral cases, and to that of the most recently conducted hearing test in bilateral cases. The patients were treated with oral antibiotics, or pus was aspirated from the middle ear when OME or AOM was not successfully treated by oral antibiotics.

The chi-squared test or Fisher’s exact test was performed for categorical variables and Mann-Whitney U test was performed for continuous variables using SPSS 22.2 for Windows (IBM Corp., Armonk, NY, USA). The results were considered statistically significant at *p* < 0.05.

The study was approved by the Institutional Review Board of Konkuk University Medical Center (KUH1110082). Because this is a retrospective study using fully anonymized data, the Institutional Review Board waived the requirement for informed consent.

## Results

In 34 adult patients with AOM or OME without dizziness, 30 patients had unilateral effusion (11 with AOM and 19 with OME) and 4 patients had bilateral effusions (2 with AOM and 2 with OME). The age range was divided into three categories as 20–40 years, 41–60 years and 61–80, the incidence of OME and AOM was compared according to the patients’ age. In 21 patients with OME, 4 patients were within the age range of 20–40 years, 7 patients were within the age range of 41–60 years, and 10 patients were within the age range of 61–80 years. In 13 patients with AOM, 7 patients were within the age range of 20–40 years, 1 patients were within the age range of 41–60 years, and 5 patients were within the age range of 61–80 years (Table 1). The distribution of patients according to age was not significantly different between OME and AOM (*p* = 0.067, chi-squared test).

**Table 1:** Age distribution of OME and AOM patients.

	OME (*n* = 21)	AOM (*n* = 13)
20–40 years	4	7
41–60 years	7	1
61–80 years	10	5

OME, otitis media with effusion; AOM, acute otitis media.

The period from symptom onset to the examination of hearing and nystagmus was 31.0 ± 40.4 days in OME and 3.5 ± 3.7 days in AOM, which was significantly different (*p* < 0.001, Mann—Whitney U test). Among 38 ears with AOM or OME, 29 showed conductive hearing loss (9 with AOM and 20 with OME), and 9 showed mixed hearing loss (6 with AOM and 3 with OME) (Table 2), and the distribution of types of hearing loss was not significantly different between AOM and OME (*p* = 0.115, Fisher’s exact test).

**Table 2:** Patterns of hearing loss according to the diagnosis of middle ear effusion.

N = 38 ears	AOM (*n* = 15)	OME (*n* = 23)
CHL (*n* = 29)	9	20
MHL (*n* = 9)	6	3

AOM, acute otitis media; OME, otitis media with effusion; CHL, conductive hearing loss; MHL, mixed hearing loss.

**Fig 1:**
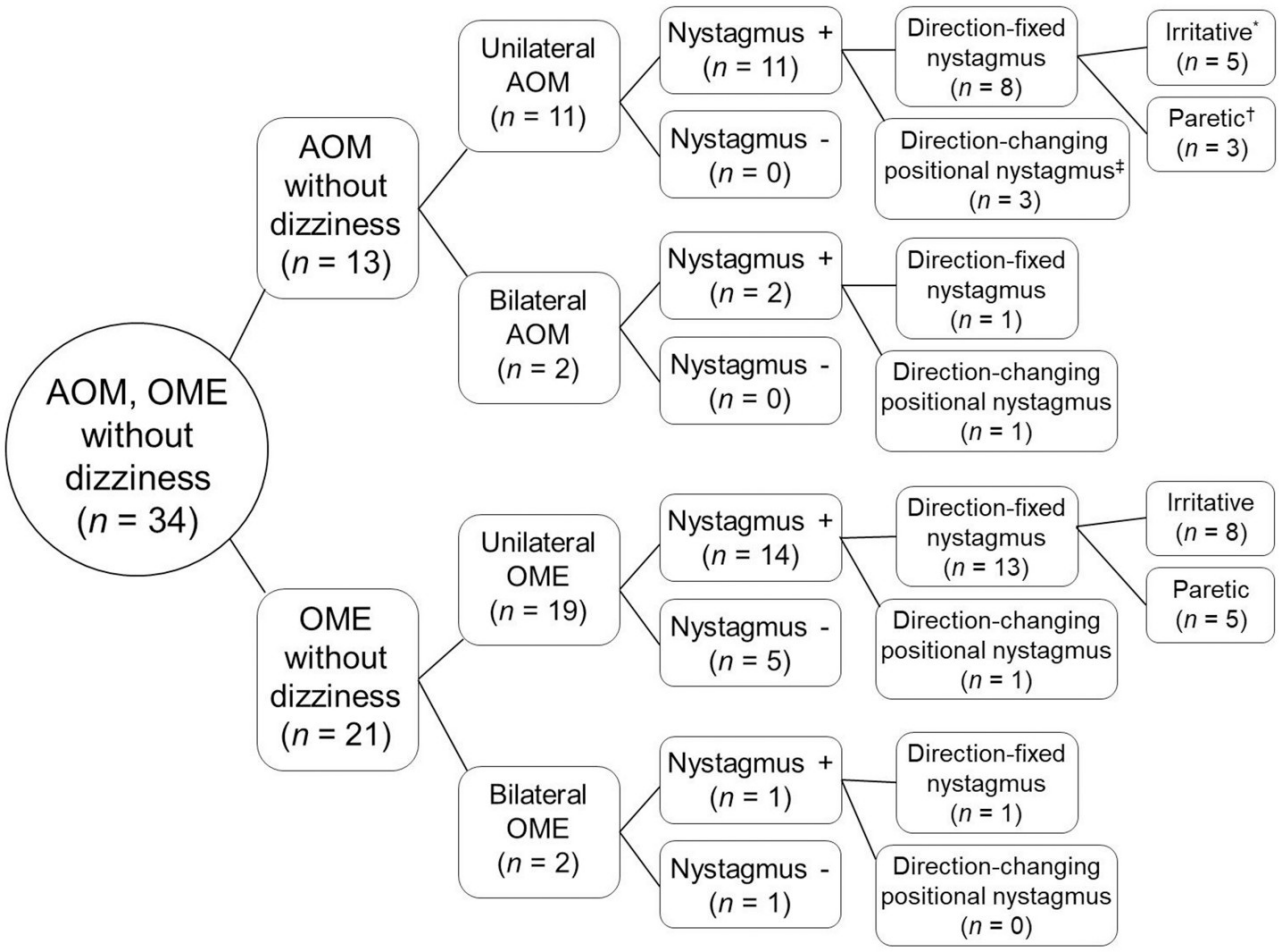
A diagram demonstrating the classification of nystagmus in adult patients of AOM or OME without dizziness (*n* = 34). *Irritative-type nystagmus beats toward the lesioned ear. ^†^Paretic-type nystagmus beats toward the healthy ear. ^‡^Direction-changing positional nystagmus was indicated when the direction of nystagmus was changed from that of spontaneous nystagmus by positioning maneuvers.

Nystagmus was observed in all of 13 AOM patients without dizziness and 15 of 21 OME patients without dizziness (Table 3, Fig 1), and the proportion of the patients with nystagmus was not statistically different between AOM and OME (*p* = 0.062, Fisher’s exact test).

**Table 3:** Number of patients showing nystagmus in patients with AOM or OME without dizziness.

	Nystagmus +	Nystagmus -
AOM (*n* = 13)	13	0
OME (*n* = 21)	15	6

AOM, acute otitis media; OME, otitis media with effusion.

In 4 patients with bilateral effusions, 2 patients showed bilateral CHL, and 2 patients showed bilateral MHL. Nystagmus was observed in 21 of 27 patients with CHL, and 7 of 7 patients with MHL. The incidence of the presence of nystagmus was not significantly different between the patients with CHL and MHL (*p* = 0.306, Fisher’s exact test).

Out of 11 unilateral AOM patients, 8 exhibited direction-fixed nystagmus, which was irritative type in 5 patients and paretic type in 3 patients. Irritative-type direction-fixed nystagmus was indicated when the observed nystagmus beats toward the lesioned ear, and paretic-type direction-fixed nystagmus was indicated when the direction of nystagmus was toward the healthy ear. Three patients exhibited direction-changing positional nystagmus, which showed different characteristics according to the patient (Table 4). Patient 1 in Table 2, who was diagnosed with AOM on the left side, exhibited weakly right-beating spontaneous nystagmus, and left-beating bowing nystagmus and right-beating leaning nystagmus were observed in a bow and lean test (S1 Video). In a head-roll test, the right head-roll evoked weak left-beating nystagmus, and the left head-roll evoked weak left-beating nystagmus with the left torsional component (S1 Video). Patient 2, who was diagnosed with AOM on the right side, showed left-beating spontaneous nystagmus with weak intensity, and both bowing and leaning nystagmus beat toward the left side. Left-beating nystagmus was observed in the right head-roll position, and weakly right-beating nystagmus with the left torsional component was observed in the left head-roll position (Table 4). Patient 3, who was diagnosed with AOM on the right side, exhibited right-beating bowing nystagmus and left-beating leaning nystagmus, and left-beating nystagmus was observed in both head-roll positions (Table 4). Of two bilateral AOM patients without dizziness, direction-fixed and direction-changing positional nystagmus were observed in one patient each (Fig 1). Patient 4 in Table 2, who was diagnosed with bilateral AOM, showed weakly left-beating spontaneous nystagmus (S2 Video). A bow and lean test evoked left- and down-beating nystagmus in a bowing position, and left-beating nystagmus in a leaning position. A head-roll test elicited weakly up-beating nystagmus in both head-roll positions (S2 Video).

**Table 4:** The patterns of direction-changing positional nystagmus in adult patients with acute otitis media (AOM) or otitis media with effusion (OME) without dizziness (*n* = 5).

No.	Sex/Age	Diagnosis	Side	Spontaneous nystagmus	Bow and lean test		Head-roll test	
					Bow	Lean	Right	Left
1	F/27	AOM	Left	RB	LB	RB	LB	LB+LT
2	M/38	AOM	Right	LB	LB	LB	LB	RB+LT
3	F/76	AOM	Right	No	RB	LB	LB	LB
4	F/78	AOM	Both	LB	LB+DB	LB	UB	UB
5*	F/77	OME	Left	No	LB	RB	LB	RB

RB, right-beating; LB, left-beating; LT, left-torsional; DB, down-beating; UB, up-beating.

*This patient was diagnosed with diffuse large B cell lymphoma in the left external auditory canal and underwent radiotherapy 1 year prior to the onset of OME in the left side.

Nystagmus was observed in 14 of 19 unilateral OME patients without dizziness (Fig 1). Among them, 13 exhibited direction-fixed nystagmus, which was irritative type in 8 patients and paretic type in 5 patients. One patient exhibited direction-changing positional nystagmus. Patient 5 in Table 4, who was diagnosed with OME on the left side after radiotherapy due to diffuse large B cell lymphoma in the left external auditory canal, exhibited weakly left-beating nystagmus in a bowing position and right-beating nystagmus in a leaning position. A head-roll test elicited ageotropic positional nystagmus with weak intensity (Table 4). Out of 2 bilateral OME patients without dizziness, one showed direction-fixed nystagmus (Fig 1).

Only two patients with unilateral AOM agreed to conduct a bithermal caloric test because the patients in this study did not complain of dizziness. One patient exhibited no unilateral weakness, and the other patient showed a canal paresis of 26% on the affected ear. During the same period (from February 2018 to November 2018), two patients with AOM, who complained of vertigo, were excluded from the study, and both of them exhibited geotropic direction-changing positional nystagmus in a head-roll test.

We also investigated spontaneous and positional nystagmus in 34 age- and sex-matched controls who have no symptom of dizziness, or history of neurologic or otologic disorders. Either spontaneous or positional nystagmus was not observed in 32 of 34 (94%) volunteers. One healthy volunteer, who is a 58 year-old man, showed weakly right-beating spontaneous and positional nystagmus, and another volunteer, who is a 28 year-old woman, showed weakly left-beating spontaneous and positional nystagmus (Table 5). The proportion of the patients with nystagmus was significantly different between patients with AOM or OME and control subjects (p < 0.001, chi-squared test).

**Table 5:** Number of patients showing nystagmus in control subjects and patients with AOM or OME.

	Nystagmus +	Nystagmus -
AOM or OME (*n* = 34)	28	6
Control (*n* = 34)	2	32

AOM, acute otitis media; OME, otitis media with effusion.

## Discussion

Although spontaneous nystagmus may be observed in healthy subjects without dizziness [25, 26], the presence of nystagmus may be a clinical sign of vestibular disturbance. However, in the present study, spontaneous or positional nystagmus was not observed in most of healthy controls (94%). Recent systematic review reported that nystagmus was present in 36% of OME patients, 93% of AOM patients, and 56% of patients with chronic otitis media [27]. The aim of the present study focused on the presence of nystagmus in AOM or OME patients without dizziness, and nystagmus was observed in most of AOM or OME patients even if they did not complain of dizziness, which was the most interesting finding of the present study.

Our results demonstrate that 28 (82%) of 34 adult patients with AOM or OME without dizziness showed nystagmus with various patterns. While nystagmus was present in all of the AOM patients without dizziness (13 of 13), 71% (15 of 21) of OME patients without dizziness exhibited nystagmus. Moreover, while the pattern of observed nystagmus was direction-changing positional nystagmus in only 7% (1 of 15) of OME patients, 31% (4 of 13) of AOM patients showed direction-changing positional nystagmus. Among unilateral AOM or OME patients showing nystagmus (*n* = 25), irritative-type direction-fixed nystagmus was the most common pattern (*n* = 13), followed by paretic-type direction-fixed nystagmus (*n* = 8), and direction-changing positional nystagmus (*n* = 4). Furthermore, the finding that all of the patients with MHL (7 or 7) showed nystagmus, and as many as 78% of the patients with CHL (21 of 27) also exhibited nystagmus was notable in the present study.

A systematic review study has demonstrated that most patients with AOM or OME, who have nonspecific vestibular symptoms or no vestibular symptoms, may have abnormal vestibular function tests [27]. The present study further revealed that OME or AOM patients without dizziness exhibited both irritative- or paretic-type direction-fixed nystagmus and direction-changing positional nystagmus. These various patterns of nystagmus have also been observed in various inner ear disorders such as Meniere’s disease, Ramsay Hunt syndrome, and sudden sensorineural hearing loss [23, 28–30]. In AOM or OME, inflammatory mediators in the middle ear may penetrate into the inner ear space through the natural membranous barriers, which may cause damages to the hair cells or blood-perilymph barrier [3, 31]. Animal experiments using mouse AOM model demonstrated that AOM induced dysfunction of blood-perilymph barrier, significantly increasing vascular permeability and leakage of serum albumin into the inner ear fluids [32]. In addition, histopathological studies have demonstrated that AOM and OME may result in significant loss of cochlear and vestibular hair cells [33, 34]. We assume that direct damage to the hair cells by the penetrated inflammatory mediators and/or biochemical changes in the inner ear fluids due to damaged blood-perilymph barrier may result in the presence of nystagmus in patients with AOM and OME. This is, as far as we know, the first report showing nystagmus findings of adult AOM or OME patients without dizziness, which highlights that various patterns of nystagmus including direction-fixed and direction-changing positional nystagmus can be observed in these patients. The presence of nystagmus, which is one of the representative signs of vestibular dysfunction, indicates that peripheral vestibular organs may be affected by AOM or OME even in patients without dizziness.

The limitation of this study is that the proposed mechanism underlying the presence of nystagmus in AOM or OME patients without dizziness is largely speculative in the present study. In addition, the most significant shortcoming of the study was that there are many potential causes for the vestibular symptoms such as metabolic diseases that have not been assessed, and therefore these variables were not adequately controlled in the current data. Furthermore, although the study suggested that AOM and OME results in nystagmus even in asymptomatic patients, it is not possible to directly correlate these factors in the present study due to the lack of further control of these confounding variables and also because of the study design. Animal studies and further clinical studies with a more profound controlling of variables could shed additional light on some of the yet unanswered questions relating otitis media and vestibular impairment.

## Conclusion

The most interesting finding of the present study was that as many as 82% (28 of 34) of AOM or OME patients exhibited nystagmus even though they did not complain of dizziness. Irritative-type direction-fixed nystagmus was the most commonly observed type, and direction-changing positional nystagmus was atypical. Direct effect of inflammatory mediators and change in chemical composition in the inner ear fluids may result in the presence of nystagmus in these patients.

## Supporting information

S1 Data (XLSX)

S1 Video Representative case of unilateral AOM showing direction-changing positional nystagmus. (AVI)

S2 Video Representative case of bilateral AOM showing direction-changing positional nystagmus. (AVI)
